# Hodentumoren aus klinischer Sicht: Was Urolog:Innen und Onkolog:Innen von Patholog:Innen über Hodentumoren wissen müssen

**DOI:** 10.1007/s00292-022-01113-0

**Published:** 2022-09-26

**Authors:** Christoph Oing, Christian Daniel Fankhauser

**Affiliations:** 1 Translational and Clinical Research Institute, Centre for Cancer, Newcastle University, NE1 7RU Newcastle upon Tyne, Großbritannien; 2 Mildred Scheel Nachwuchszentrum HaTriCS4, Universitäres Cancer Center Hamburg, Universitätsklinikum Eppendorf, Martinistr. 52, 20246 Hamburg, Deutschland; 3 Klinik für Urologie, Kantonsspital Luzern, Luzern, Schweiz

**Keywords:** Keimzelltumor, Isochromosom 12p, MicroRNA 371a-3p, Syncytiotrophoblast, Seminom, Germ cell tumour, Isochromosome 12p, MicroRNA 371a-3p, Syncytiotrphoblast, Seminoma

## Abstract

**Hintergrund:**

Keimzelltumoren des Hodens sind die häufigste maligne Tumorerkrankung bei Männern im Alter von 15–40 Jahren. Die Unterscheidung von Seminomen und Nichtseminomen hat prognostische Bedeutung und ist für Therapieplanung und Nachsorge essenziell. Durch interdisziplinäre, stadiengerechte Therapie haben Keimzelltumoren generell eine sehr gute Prognose. Eine Übertherapie sollte wegen möglicher Langzeitfolgen vermieden werden. Hierbei hilft die Risikobeurteilung anhand klinischer und pathologischer Faktoren.

**Ziel der Arbeit:**

Darstellung der (histo-)pathologischen Charakteristika, die die uroonkologische Therapieplanung maßgeblich beeinflussen.

**Material und Methoden:**

Nichtsystematischer Übersichtsartikel über die relevanten (histo-)pathologischen Befunde für die klinische Therapieplanung im interdisziplinären Kontext.

**Ergebnisse:**

Zentrale Pathologiebefunde für Kliniker:Innen sind: (i) Identifikation eines Keimzelltumors, ggf. durch Nachweis eines Chromosom-12p-Zugewinns, (ii) Subtypenspezifizierung und (iii) Angabe von Risikofaktoren (insbesondere Invasion von Lymphgefäßen und/oder Rete testis und Tumorgröße). Molekularpathologische Untersuchungen i. S. von Mutationsanalysen sind angesichts einer sehr geringen Mutationslast und bislang fehlender prädiktiver Marker und zielgerichteter Therapieoptionen nicht Teil der Routinediagnostik.

**Diskussion:**

Ein detaillierter, idealerweise synoptischer histopathologischer Befundbericht ist Grundlage der Planung und Durchführung einer leitlinienkonformen, risikoadaptierten Therapie und neben der bildgebenden Diagnostik und der Bestimmung der Serumtumormarker AFP und β‑HCG (letztere insbesondere bei Nichtseminomen) mitentscheidend, um die guten Heilungsaussichten zu wahren und eine Übertherapie zu vermeiden.

**Zusatzmaterial online:**

Zusätzliche Informationen sind in der Online-Version dieses Artikels (10.1007/s00292-022-01113-0) enthalten.

## Abstract (en)

**Background:**

Testicular germ cell tumours (GCTs) are the most frequent solid malignancy in younger males aged 15–40. The differentiation between seminomas and non-seminomas impacts prognosis, clinical management and follow-up procedures. With stage- and risk-adapted multimodal treatment approaches, GCTs have an exceptionally good prognosis. Therefore, avoiding overtreatment to reduce treatment-related long-term side effects is of utmost importance. Clinical and histopathological risk factors aid in treatment decision-making.

**Objectives:**

Discussion of (histo-)pathological characteristics that directly influence treatment decision-making by urologists and oncologists.

**Materials and methods:**

Non-systematic literature review to describe histopathological features for interdisciplinary treatment planning.

**Results:**

Key histopathological characteristics for clinicians are: (i) identification of a GCT, if necessary by 12p aberration analysis, (ii) description of the different subtypes, and (iii) risk factors, including lymphovascular invasion and/or rete testis infiltration and size of the primary tumour. Molecular pathological analyses, that is, genomic sequencing, is not part of routine diagnostics due to the lack of prognostic/predictive markers and effective targeted treatment approaches.

**Discussion:**

Detailed histopathology reporting, ideally with a synoptic template, is the basis for planning and conducting guideline-endorsed, risk-adapted, multi-disciplinary management of GCTs. Along with radiographic imaging and assessment of the serum tumour markers AFP and β‑HCG (especially in non-seminomas), histopathology is crucial to maintain success and reduce the burden of GCT treatment.

Keimzelltumoren (KZT) des Mannes sind biologisch besonders und zeigen eine hohe Sensitivität gegenüber Chemo- und/oder Radiotherapie (mit Ausnahme reifer Teratome) und exzellente Heilungsaussichten selbst in metastasierten Stadien. Neben der Stadieneinteilung gemäß *Union for International Cancer Control *(UICC) und der Risikoklassifikation der *International Germ Cell Cancer Collaborative Group* (IGCCCG) für alle bzw. fortgeschrittene Stadien, ist die Kenntnis weiterer KZT-spezifischer, stadienbezogener Risikofaktoren aus der (histo-)pathologischen Befundung für die klinische Behandlungsplanung unabdingbar. Schnittstellen der Interaktion zwischen Pathologie, Urologie und Onkologie werden nachfolgend beschrieben.

## Epidemiologie

Mit knapp 4000 Neuerkrankungen im Jahr in Deutschland zählen Keimzelltumoren (KZT) des Mannes zu den selteneren soliden Tumorerkrankungen in Deutschland [1]. Dennoch sind KZT das häufigste solide Malignom bei Männern zwischen 15 und 40 Jahren [2]. Deutschland und die Schweiz haben eine hohe Inzidenz im weltweiten Vergleich mit 10,0 bzw. 9,4/100.000 Einwohnern im Jahr [3]. Wichtigste Risikofaktoren für die Entstehung eines malignen KZT des Hodens sind ein vorheriger kontralateraler Hodentumor, KZT-Erkrankungen bei Verwandten ersten Grades und ein Maldescensus testis bzw. Kryptorchismus [4].

Das krebsspezifische Überleben ist über alle Stadien hinweg exzellent. Jährlich sterben in Deutschland lediglich ca. 150 Patienten an einem KZT [1]. Die hohen Heilungsraten sind insbesondere der oft frühen Diagnosestellung in lokalisierten Stadien und einer ausgesprochenen Sensitivität gegenüber cisplatinbasierter Chemotherapie in fortgeschrittenen Stadien zuzuschreiben [5].

## Pathologische Untersuchung des Hodengewebes und weiterer Gewebeproben

Circa 95 % der männlichen KZT entstehen im Hodenparenchym, 5 % dagegen primär extragonadal, d. h. retroperitoneal, mediastinal oder im Mittelhirn (hypothalamisch-hypophysär) [6]. Knapp 60 % aller KZT sind reine Seminome, 40 % Nichtseminome mit meist gemischten KZT-Anteilen [7].

Aktuelle Leitlinien (u. a. S3, EAU und ESMO) definieren Anforderungen an die histopathologische Aufarbeitung von Hodengewebeproben nach inguinaler Orchidektomie [8–11]. Idealerweise werden die Elemente als synoptischer Report aufgelistet, sodass eine komplette Berichterstattung garantiert wird und die Befunde unmittelbar ersichtlich sind (siehe Supplementary table 1).

Neben diesen klassischen Befundcharakteristika ist die Kenntnis weiterer pathologischer Faktoren insbesondere in metastasierten Erkrankungsstadien und/oder extragonadaler Primärmanifestation entscheidend für das Patientenmanagement [11]:

1. Detektion synzytiotrophoblastärer Riesenzellen und bestmöglicher Ausschluss anderer, nichtseminomatöser Keimzelltumoranteile (insb. Chorionkarzinom) bei reinen Seminomen zur Erklärung mitunter exzessiver Erhöhung der β‑Untereinheit des humanen Choriongonadotropins (β-HCG).
2. Nachweis eines Zugewinns genomischen Materials von Chromosom 12p (Isochromosom oder andere Amplifikationen) zum Nachweis eines Keimzelltumors als Ursprung eines malignen Tumors, insb. bei unklarer Linienzugehörigkeit oder Verdacht auf somatische Malignität auf Basis eines Teratoms.
3. Detaillierte Beschreibung der histologischen Tumorkomposition und relativer Anteile möglicherweise vorhandener unterschiedlicher Gewebe in resezierten postchemotherapeutischen Residualtumoren (Nekrose/Fibrose versus vitaler Keimzelltumor versus Teratom).

Die klinische Bedeutung vorgenannter Faktoren werden im Folgenden aus uroonkologischem Blickwinkel diskutiert.

## Histopathologische Risikofaktoren bei testikulären Keimzelltumoren im Stadium I

Männliche KZT werden vorwiegend im klinischen Stadium I, also lokalisiert im Hoden diagnostiziert [12]. Das krebsspezifische Überleben für diese Patienten beträgt nach radikaler inguinaler Orchiektomie für Seminome wie Nichtseminome > 99 % (ungeachtet adjuvanter Therapiemaßnahmen) [8, 10].

### Seminom

Eine zunehmende Primärtumorgröße und/oder der Nachweis einer Rete-testis-Infiltration erhöhen das Risiko einer okkulten Metastasierung von ca. 15 % auf 20 % bis > 30 % bei Nachweis von 1 bzw. 2 Risikofaktoren [13]. Die Primärtumorgröße ist der bedeutendste Prognosefaktor, wohingegen die Rolle der Rete-testis-Invasion weniger evident ist [14]. Ungeachtet möglicher Risikofaktoren wird für alle Seminome im Stadium I die aktive Überwachung nach inguinaler Orchiektomie als bevorzugte Maßnahme empfohlen [8–10]. Sollte ein Patient zusätzliche Maßnahmen zur Verringerung des Rezidivrisikos wünschen, kommt eine adjuvante Chemotherapie (1 Zyklus Carboplatin AUC7) oder eine Radiatio der ipsilateralen, paraaortalen Lymphabflusswege mit kumulativ 20 Gy infrage. Beide Maßnahmen reduzieren das Rückfallrisiko auf < 5 %, ohne jedoch das exzellente Gesamtüberleben zusätzlich zu verbessern [9, 10].

### Nichtseminom

Die lymphovaskuläre Invasion (LVI) ist der wichtigste Risikofaktor, bei deren Nachweis das Risiko für eine okkulte retroperitoneale Metastasierung 50 % beträgt (ohne LVI ca. 15–20 %) [15, 16]. Auch eine Rete-testis-Invasion oder Einbezug hilärer Gewebeanteile erhöhen das Metastasierungsrisiko und sollten histopathologisch begutachtet werden, wenngleich die Evidenz hierfür geringer ist [17]. Durch adjuvante Gabe eines Zyklus Cisplatin, Etoposid und Bleomycin (PEB) wird das Rezidivrisiko auf ca. 3 % reduziert [18]. 1 Zyklus PEB ist damit für LVI-positive Patienten generell empfohlen, da im Rezidivfall in der Regel 3–4 Zyklen PEB ggf. gefolgt von einer chirurgischen Residualtumorresektion zur Therapie einer metastasierten Erkrankung erforderlich werden. Die alternative adjuvante retroperitoneale Lymphadenektomie bietet den Vorteil des genauen histopathologischen Stagings, ist aber aufgrund der Abhängigkeit des Erfolgs und der therapieassoziierten Morbidität von der Erfahrung des Zentrums/Operateurs in den Hintergrund gerückt [8, 9, 11].

## β-HCG-produzierende Seminome

Knapp 20 % der Seminompatienten zeigen eine Erhöhung des β‑HCG-Serumspiegels [8, 10, 19]. Diese übersteigen selten Werte von 500 IU/L, können mitunter aber auch deutlich höher ausfallen. Bei sehr hohen β‑HCG-Werten wird ein reines Seminom als Diagnose häufig bezweifelt, weshalb gerade in solchen Situationen die ausführliche histopathologische Aufarbeitung bedeutsam ist (insbesondere um nichtseminomatöse Chorionkarzinomanteile auszuschließen) [11].

**Figure 1:**
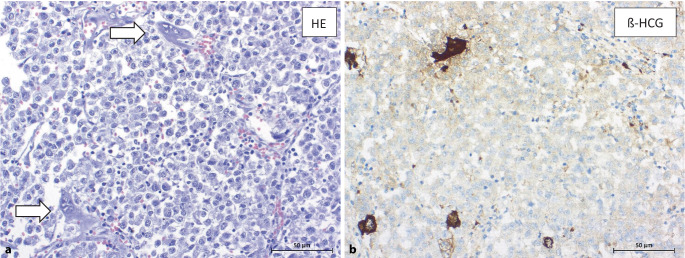

Obwohl die Serumtumormarker in der IGCCCG-Klassifikation für das Seminom bisher keine Rolle spielen, sind deutlich erhöhte β‑HCG-Serumspiegel (≥ 2000 IU/L) beim Seminom prognostisch ungünstig [20] und i. d. R. auf synzytiotrophoblastäre Riesenzellen zurückzuführen. Als Erklärung erhöhter β‑HCG-Serumspiegel sollte versucht werden, diese zu identifizieren und zu berichten [21]. Siehe hierzu auch Abb. 1 aus Oing et al. [11]. Der Nachweis synzytiotrophoblastärer Riesenzellen kann damit helfen, Zweifel am Vorhandensein eines nichtseminomatösen Keimzelltumors mit Chorionkarzinomanteilen auszuräumen. Neben der β‑HCG-Erhöhung zeigte sich auch eine Erhöhung der LDH (≥ 2,5faches der oberen Norm) als negativer Prädiktor für das Therapieansprechen und Gesamtüberleben [22, 23]. Ob dies allein der hohen Tumorlast oder distinkten biologischen Eigenschaften zuzuschreiben ist, bleibt bislang offen [11].

## MicroRNA miR-371a-3p

Die klassischen Serumtumormarker β‑HCG, AFP und LDH helfen bei Diagnosestellung, Beurteilung des Therapieansprechens und im Rahmen der Tumornachsorge. Problematisch ist die unzureichende Sensitivität mit > 50 %, sodass falsch negative Befunde keine Seltenheit sind und Teratome diese generell nicht exprimieren [24].

Zwei miR-Cluster, miR-371-373 und miR-302/367, wurden als neue, KZT-spezifische Biomarker identifiziert, wovon miR-371a-3p die höchste Sensitivität (90,1 %) und Spezifität (94,1 %) aufwies [25, 26]. Die miR-371a-3p wird vorwiegend in KZT-Zellen, aber auch in der Vorläuferläsion „germ cell neoplasia in situ“ (GCNIS) und geringgradig auch in gesundem Hodenparenchym exprimiert [27]. Die Spiegel in KZT-Gewebe und Patientenserum korrelieren signifikant [28]. Entsprechend korreliert auch die Höhe des miR-371a-3p-Serumspiegels mit der Tumormasse und folglich dem klinischen Stadium [26]. Dies gilt allerdings nicht für reife Teratome, die leider keine miR-371a-3p exprimieren. Weitere miRNA-Signaturen, z. B. für die Unterscheidung zwischen lokalisierten und metastasierten Seminomen, befinden sich in klinischer Evaluation [29].

Die miR-371a-3p eignet sich angesichts der hohen Sensitivität und Spezifität und einer kurzen Serumhalbwertszeit wahrscheinlich besser zum Nachweis einer aktiven (nichtteratomatösen) KZT-Erkrankung als die konventionellen Serumtumormarker und damit u. a. (i) zur Überwachung eines Therapieansprechens, (ii) zur frühzeitigen Detektion von okkulten Metastasen in frühen klinischen Stadien, (iii) vitalen KZT-Residuen nach Chemotherapie oder (iv) der frühestmöglichen Detektion von Rezidiven nach erfolgreicher multimodaler Therapie. Bislang ist die Bestimmung der miR-371-3p oder anderer miRNA-Cluster jedoch nicht Teil der Routinediagnostik. Zunächst erfolgt der Einsatz in klinischen Studien, um die qRT-PCR-basierte miR-371a-3p-Diagnostik mit ausreichender Evidenz zu untermauern.

## Chromosom-12p-Alterationen

**Figure 2:**
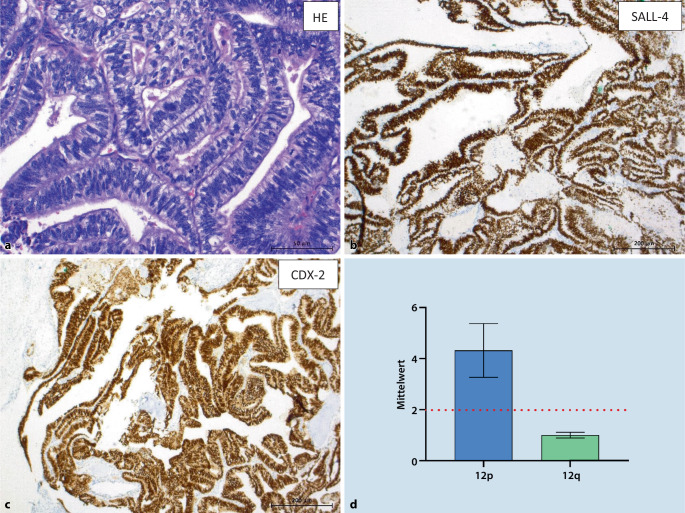

Aberrationen des kurzen Arms von Chromosom 12 markieren den Übergang einer GCNIS in einen invasiven KZT [30]. Das Isochromosom 12p (i[12p]) ist bei bis zu 89 % aller KZT nachweisbar, bei i(12p)-negativen Tumoren findet man meist regionale 12p-Amplifikationen [6, 31, 32]. Durch den Nachweis lässt sich ein KZT sicher identifizieren, was insbesondere bei der Zuordnung metastastischer Läsionen unklarer Herkunft („cancer of unknown primary“, CUP) und somatischer Malignität eines Teratoms oft der einzige Hinweis auf die Grunderkrankung ist (Abb. 2). Der Nachweis eines i(12p) erfolgt per Fluoreszenz-in-situ-Hybridisierung [33] oder PCR-basiert [34]. Letzteres Verfahren ist zeitsparender, kostengünstiger und erfasst auch andere 12p-Aberrationen und damit einen KZT-Ursprung auch bei den wenigen i(12p)-negativen KZT [34]. Die Auswahl der Primärtherapie richtet sich auch bei Prädominanz einer somatischen Malignität nach den Empfehlungen zur Behandlung von KZT und lässt die bestimmende Histomorphologie damit zunächst außer Acht [9, 35].

## Postchemotherapeutische Residualtumorresektion

Postchemotherapeutische Residualtumormanifestationen zeigen sich bei ca. 1/3 aller metastasierten KZT-Erkrankungen [36]. Das therapeutische Vorgehen unterscheidet sich maßgeblich je nach Histologie der Grunderkrankung.

### Seminom

Residualtumoren < 3 cm sind in aller Regel nekrotisch/fibrotisch. Erst bei Residuen ≥ 3 cm finden sich ggf. auch vitale Seminomanteile [37]. Für die Vitalitätsbeurteilung kann bei Residuen ≥ 3 cm eine Positronen-Emissions-Tomograhie/Computertomographie (PET-CT) hilfreich sein, die zur Vermeidung falsch positiver Befunde frühestens 6–8 Wochen nach Ende der Chemotherapie durchgeführt werden sollte [38]. PET-negative Residuen werden nachbeobachtet, für PET-positive Läsionen ist das optimale Management bislang unzureichend definiert [39]. Als Alternative zur teils aufwendigen operativen Resektion kann eine kurzfristige CT-graphische Verlaufskontrolle nach interdisziplinärer Diskussion erwogen werden [9].

### Nichtseminom

Das Residualtumormanagement beim Nichtseminom ist durchaus aggressiver, da Residuen ≥ 1 cm deutlich häufiger vitale KZT-Anteile (10–15 %) und/oder chemotherapieresistente Teratomanteile (40–45 %) enthalten [36]. Residuen > 1 cm nach Erstlinienchemotherapie sollten daher bei Normalisierung oder Stabilisierung erhöhter Serumtumormarker, sofern technisch möglich, operativ reseziert werden. Dies gilt insbesondere für Residualtumoren in Retroperitoneum und Lunge. Bei anderen viszeralen Metastasen ist das Prozedere von der Resektabilität, der erwarteten Morbidität und der Histologie der zuvor resezierten Residualtumoren abhängig [9]. Bei ausgedehnter Metastasierung mit ungünstigem IGCCCG-Risikoprofil und prädominanten Teratomanteilen im gonadalen Primärtumor sollte auch eine Resektion retroperitonealer Residuen < 1 cm erwogen werden [40]. Steigen vor geplanter Residualtumorresektion die Serumtumormarker, sollte zunächst eine Salvage-Chemotherapie appliziert und anschließend, sofern technisch möglich, alle sichtbaren Residuen reseziert werden, da häufiger platinrefraktäre KZT-Anteile gefunden werden [9].

Die histopathologische Befundung postchemotherapeutischer Residuen sollte unbedingt eine relative Quantifizierung vitaler KZT-Anteile berichten, denn diese hat klinische Relevanz. Beträgt der Anteil vitaler KZT-Zellen ≥ 10 % der Residualtumormasse kann eine zusätzliche adjuvante cisplatinbasierte Chemotherapie möglicherweise die Heilungsraten verbessern [36, 41–43].

## Platinrefraktäre Keimzelltumoren

Nach cisplatinbasierter Kombinationschemotherapie erleiden ca. 15–30 % der Patienten mit metastasierter KZT-Erkrankung ein Rezidiv [19]. Multimodale Salvage-Therapien inklusive hochdosierter Chemotherapie mit autologer Stammzelltransplantation erreichen immerhin noch Heilungsraten von 50 % [44]. Patienten, die wiederholte Rezidive oder einen Erkrankungsprogress während cisplatinbasierter Chemotherapie erleiden, gelten als platinrefraktär und haben eine sehr ungünstige Prognose [45]. Eine Bestimmung molekularpathologischer Marker außerhalb klinischer Studien hat bisher keine Bedeutung in der Therapieplanung. Aufgrund einer durchweg geringen Tumormutationslast [46] und einer geringen Inzidenz targetierbarer Treibermutationen sind auch genomische Sequenzierungsverfahren bislang wenig hilfreich [11]. Die häufigsten Aberrationen in platinrefraktären KZT betreffen *TP53, KRAS, NRAS* und *c‑KIT* neben möglicherweise künftig therapierbaren Aberrationen im Wnt/ß-Catenin- und PI3K/AKT/MAPK-Signalweg [47, 48].

Zahlreiche frühe klinische Studien konnten keine relevante Wirksamkeit u. a. für Tyrosinkinaseinhibitoren (z. B. Sunitinib, Pazopanib, Imatinib, Sorafenib), Immuncheckpointinhibitoren (Pembrolizumab, Avelumab, Durvalumab ± Tremelimumab) oder Antikörper-Wirkstoff-Konjugate (Brentuximab-Vedotin) zeigen [11, 49, 50]. Wahrscheinliche Ursachen für die fehlende Wirksamkeit sind (i) die ausgeprägte Heterogenität der KZT, (ii) das Fehlen prädiktiver Biomarker für zielgerichtete Therapiestrategien und damit (iii) eine unzureichende Patientenselektion [49]. Die systemische Behandlung fortgeschrittener KZT bleibt somit zunächst eine Domäne der konventionellen Zytostatikatherapie [11, 46–48].

Mit Spannung werden zudem Ergebnisse der frühen klinischen Studien zum Einsatz modifizierter T‑Zellen mit chimären Antigenrezeptoren (CAR-T) und/oder Vakzinierungsstrategien bei soliden Tumoren erwartet. In sog. Basket-Studien konnten z. B. auch Keimzelltumorpatienten eingeschlossen werden, die bestimmte Oberflächenantigene als Zielstrukturen exprimierten, z. B. „preferentially expressed antigen in melanoma“ (PRAME; NCT03686124) oder Claudin‑6 (NCT04503278). Die Untersuchung auf mögliche Zielstrukturen für neuartige zellbasierte Immuntherapieverfahren sollte jedoch auch nur als Screeningmaßnahme an Zentren, die entsprechende Studien durchführen, erfolgen.

## Fazit für die Praxis

- Eine fundierte histopathologische Befundung ist für die Therapieplanung und Beurteilung des Therapieansprechens essenziell.
- Synzytiotrophoblastäre Riesenzellen in Seminomen können teils deutliche β‑HCG-Erhöhungen erklären und helfen, Chorionkarzinomanteile auszuschließen.
- Ein Zugewinn genetischen Materials von Chromosom 12p (insb. i[12p]) ist prognostisch und therapeutisch relevant. Es definiert einen Keimzelltumorursprung bei Tumoren unklarer Herkunft bzw. Differenzierung.
- MicroRNA miR-371a-3p ist ein hochspezifischer und -sensitiver Tumormarker (Teratome ausgenommen), der zunächst außerhalb klinischer Studien noch nicht in Diagnostik, Therapieplanung oder Nachsorge genutzt werden sollte.
- Prädiktive Biomarker für zielgerichtete Therapiekonzepte bei platinrefraktärer Erkrankung existieren nicht und so spielen genomische Sequenzierungen außerhalb von Studien bislang keine Rolle.

## Supplementary Information




