# Supplementary material for: Testicular tumours from a clinical point of view: What urologists and oncologists need to know from the pathologist about testicular cancer
Source: Pathologie (Heidelb). 2022 Sep 26;43(6):434–40. [Article in German] doi: 10.1007/s00292-022-01113-0 (PMC9585009; doi:10.1007/s00292-022-01113-0)
Supplement: Supplementary file 1 [file 292_2022_1113_MOESM1_ESM.docx]

**Tabelle 1:**

| 1. | Betroffene Seite | Links/rechts/bilateral |
| --- | --- | --- |
| 2. | Größe des Hodens, gesamt (3 Dimensionen) | [cm] |
| 3. | Maximale Tumorgröße (3 Dimensionen) | [cm] |
| 4. | Makroskopische Merkmale   - Nebenhoden - Rete Samestrang - Tunica vaginalis |  |
| 5. | Tumor im Absetzungsrand | ja/nein |
| 6. | Histologischer Typ mit Subspezifizierung individueller Komponenten und prozentualer Bestimmung gemäß aktuell gültiger WHO Klassifikation |  |
| 7. | Peritumorale venöse und/oder lymphatische Invasion | ja/nein |
| 8. | Invasion angrezender Strukturen   - Tunica albuginea - Tunica vaginalis - Rete testis - Weichgewebe des Hilus - Weichgewebe des Nebenhodens oder Samenstranges | ja/nein |
| 9. | GCNIS im nicht-tumorösen Parenchym | ja/nein |
| 10. | pT-Kategorie gemäß aktueller TNM-Klassifikation |  |
| GCNIS, *Germ cell neoplasia in situ* | | |

**Supplementary table 1:** Histopathologische Charakterisitka für die Leitlinien-konforme Primärtumorbeurteilung nach radikaler inguinaler Orchiektomie [8-10].
